# The Mental Landscape of Imagining Life Beyond the Current Life Span: Implications for Construal and Self-Continuity

**DOI:** 10.1093/geroni/igaa013

**Published:** 2020-06-27

**Authors:** Brittany M Tausen, Attila Csordas, C Neil Macrae

**Affiliations:** 1 School of Psychology, Seattle Pacific University, Washington; 2 Open Lifespan, Cambridge, UK; 3 School of Psychology, University of Aberdeen, UK

**Keywords:** Aging, Future, Life extension, Prospection, Self-continuity, Temporal distance

## Abstract

**Background and Objectives:**

With rapid advancements in medicine, technology, and nutrition, the future holds the possibility of longer and healthier lives. Despite garnering attention from myriad disciplines, psychological perspectives on life extension are scarce. In three studies, we addressed this gap by exploring key mental characteristics and psychological variables associated with simulating an expanded life span and thus an extremely distant future self.

**Research Design and Methods:**

Three studies investigated the construal (i.e., valence, vividness, and visual perspective) of extremely distant future simulations and the extent to which participants felt connected to their future selves (i.e., self-continuity). Studies 1 and 2 investigated the characteristics of imagery associated with different ages ranging from near the current species maximum (e.g., 120, 150) to more highly hypothetical ages (e.g., 201, 501). Study 3 probed the mental construal of extreme aging among different populations (i.e., life-extension supporters, students, and Mechanical Turk workers). Studies also assessed participants’ general feelings about the ethicality and likelihood of techniques that halt or reverse biological aging to help individuals live beyond the current life expectancy.

**Results:**

Participants in all studies reported being able to vividly imagine expanded aging scenarios (increased chronological, without biological, and aging), but these simulations were characterized by a decreased sense of connection to one’s future self (i.e., self-continuity) compared to a control condition. Temporal distance did not, however, impact ratings of self-continuity when comparing experimental conditions (i.e., imagining one’s self 120 vs 150 or 201 vs 501). Curiously, a sense of self-continuity (when simulating oneself well beyond the current life expectancy) remained intact for individuals who belonged to a community of life-extension supporters. The perceived likelihood and ethicality of extended life-span scenarios also varied significantly across different populations.

**Discussion and Implications:**

The current work is the first to quantify the disconnect between one’s current and extremely distant (i.e., beyond the current life expectancy) future self. Given the behavioral implications of feeling disconnected from one’s future self (e.g., failing to save for retirement or care for one’s own physical health), these findings inform a critical barrier of extended life spans and provide insight into potential remedies (e.g., enhancing the perceived likelihood of living longer). Theoretical implications of hypotheticality and temporal distance, two key dimensions of Construal Level Theory, and their impact on the construal and self-continuity associated with future simulations are also discussed.

**Translational Significance:** A potential psychological barrier associated with increased life expectancy is feeling disconnected from one’s future self. The current work leveraged mental imagery to assess *when* and for *whom* this barrier is most prominent. Results inform strategies to create continuity in one’s sense of self over time, enhancing the likelihood of actualizing the benefits and minimizing the costs of living longer lives.

In a world of medical and technical advancements, extended life spans are becoming increasingly more tangible. What was once merely gripping science fiction or fodder for deep existential musings now seems to have significant scientific traction (Longo et al., 2015). Whether through uploading one’s consciousness to a machine (Bamford, 2012), or undergoing regenerative aging interventions (Romain, 2010), it is possible that the not-too-distant future holds opportunities to dramatically increase the life span (Sandberg & Bostrom, 2008). To date, regenerative aging interventions are the most widely accepted and realistic approaches to achieving extreme longevity, circumventing deeply philosophical questions about the soul and immortality. Indeed, a growing number of individuals are already engaged in variations of such interventions, from caloric restriction to pharmacological supplementation (Longo et al., 2015). While certainly a topic for moral and sustainability debates (Schneider, 2009), one cannot help but wonder what it might be like to live for hundreds of years—to have the opportunity to experience the extremely distant future. Philosophers, writers, and computer scientists have painted both enticing (Schloendrom, 2006) and distressing (Pijnenburg & Leget, 2007) pictures of life extension, but a psychological perspective remains largely absent from the literature. Aside from the ethical, environmental, and technical hurdles of life extension, one might imagine that there will also be concerns about mental health and well-being if individuals should begin living well beyond their currently expected number of years. In fact, the question remains whether or not individuals would even want to live beyond their expected life span and if so, for how long?

Inquiries about the desirability of, and attitudes associated with, extended life spans are becoming more common in the literature (see Ballinger, Tisdale, Sellen, & Martin, 2017; Bowen et al., 2019; Kogan, Tucker, & Porter, 2011). The increase in attention to life extension is representative of a global phenomenon. According to the World Health Organization, life expectancy increased 5 years between 2000 and 2015, the largest documented increase since the 1960s. For the United States and the United Kingdom, homes to some of the highest life expectancy rates, expectancies have nearly doubled since the 1900s (Pew Research Center, 2013a; Public Health England, 2016), with the number of centenarians worldwide expected to increase approximately eightfold by 2050 (Pew, 2016). From a psychological perspective, the response to these trends has been twofold: (1) consider the mental and physical changes associated with aging, and (2) investigate the cognitive and emotional components associated with the idea of living longer lives.

Paradoxically, at a time when life spans are increasing, ideas about aging are often quite negative. In particular, individuals report fears associated with loss later in life as well as the physical and psychological effects of aging (Tahmaseb et al., 2003). Indeed, research describing late adulthood has confirmed considerable physical and cognitive decrements associated with aging (Schaie & Willis, 2011). Eyesight, muscle strength, reaction times, and the ability to recall new information (to name but a few) all decline with age while the susceptibility to diseases increases (Cleary, Zaborski, & Ayanian, 2004; Myers & Dewall, 2015). Importantly, however, the picture of aging is not as bleak as it might seem. Contrary to popular belief, self-esteem, life satisfaction, and subjective well-being tend to be relatively stable across the life span (Lansford, 2018; (Jebb, Morrison, Tay, & Diener, 2019)). Late adulthood is also often characterized by increased positive affect, emotional control, stability, and acceptance (Carstensen et al., 2011; Shallcross, Ford, Floerke, & Mauss, 2013; Stone, Schwartz, Broderick, & Deaton, 2010; Urry & Gross, 2010). What is more, the most dramatic decreases in mental state and ability are not seen until the final years of life, a phenomenon referred to as terminal decline (Backman & MacDonald, 2006). Indeed, some researchers suggest that “nearness to death” is a better predictor of mental states and abilities than age (Shrira, Bodner, & Palgi, 2014). As the picture of late adulthood continues to emerge, psychologists are able to more clearly describe the process of aging and to more fruitfully consider interventions to aid an increasingly elderly population.

An additional (and also growing) body of research has focused not on the experience of being older, but thinking about being older. Prospection, the ability to transcend the present moment and contemplate future points in time, is often argued to be something that is not only unique to humans, but also highly adaptive (Moulton & Kosslyn, 2009; Suddendorf & Corballis, 2007). Exploring prospection can also be incredibly insightful, elucidating links between the conceptualization of the future self and related behaviors. According to the Construal Level Theory, temporal distance is a key dimension of Psychological Distance—a factor identified to impact the way that events are conceptualized. Investigations of imagining one’s future self have revealed that the characteristics associated with mental simulation vary as a function of temporal distance. In particular, distant experiences tend to be construed as more positive, abstract and conceptual whereas proximal experiences are often characterized by concrete and detailed mental representations (see Liberman & Trope, 1998; Trope & Liberman, 2003, 2010). Narratives describing one’s distant future (e.g., 3 years from now) self also tend to be characterized by more positive and predictable content than narratives describing one’s near future (e.g., 1 month from now) self (Heller, Stephan, Kifer, & Sedikides, 2011; Stephan, Sedikides, Heller, & Shidlovski, 2015). Curiously, the impact of age on mental construal tends to run parallel to the consequences of temporal distance, such that older adults simulate the distant future with less episodic specificity (Addis, Wong, & Schacter, 2008). In other words, their simulations tend to be more abstract and generalized (see Löckenhoff & Rutt, 2017). Beyond isolated explorations of age and temporal distance, it is important to note that the two are not orthogonal factors. When participants are asked to imagine a specific age (as will be the case in the present studies), temporal distance inherently varies as a function of one’s current age. Clearly, temporal distance and age shape the manner in which an event is mentally constructed and should be considered in tandem.

Aside from a distinct mental construal, imagining the distant future is also characterized by myriad behavioral discrepancies. Contrasting simulations of the near and distant future has revealed significant differences in procrastination behaviors (McCrea, Liberman, & Trope, 2008), willingness to delay gratification (Chapman, 1996; Chapman & Elstein, 1995; Frederick, Loewenstein, & O’Donoghue, 2002), choices to undergo difficult or unpleasant tasks, and efforts to act prosocially (Pronin, Olivola, & Kennedy, 2008). The incongruity tends to be so pronounced that decisions for one’s future self are likely to resemble decisions made for an entirely different person more than they do decisions made for the present self (Pronin et al., 2008; Pronin & Ross, 2006; Wakslak, Nussbaum, Liberman, & Trope, 2008). Thus, self-continuity, or the sense of overlap between one’s current and future self, has become a key variable of interest when exploring the otherwise puzzling differences in present versus future decision-making processes (Bartels & Rips, 2010; Ersner-Hershfield, Garton, Ballard, Samanez-Larkin, & Knustson, 2009; Ersner-Hershfield, Wimmer, & Knutson, 2009). Curiously, the visual perspective employed to imagine an event has a significant impact on one’s sense of self-continuity (Tausen, Miles, Lawrie, & Macrae, 2018). Not only can a lack of continuity manifest in the adoption of a third-person perspective, but manipulating perspective can result in feeling disconnected from one’s past or future self (see Tausen, Carpenter, & Macrae, 2019 for review). For individuals who feel more in tune with their future selves, or acknowledge stronger links between current decisions and future consequences, there is less difference between choices made for the near and distant future (Bartels & Urminsky, 2011; Hershfield, 2011; Mitchell, Schirmer, Ames, & Gilbert, 2011; Parfit, 1971, 1987; Schelling, 1984; Stephan, Shidlovoski, & Sedlikides, 2018; Thaler & Shefrin, 1981).

A systematic review article by Löckenhoff and Rutt (2017) has extended the investigations of perceived future self-continuity to an older population. The review highlights an impressive body of indirect evidence for a relationship between age and self-continuity ranging from differences in the perception of the passage of time to the increased stability of personality traits and the coherence of personal narratives for older adults (Troll, Skaff, & McKean, 1997). Even research from behavioral economics provides supporting evidence for increased self-continuity with age. Temporal discounting, in particular, is a phenomenon in which individuals assign a diminished value to a reward simply because it is delayed in time. The behavioral manifestation of temporal discounting—foregoing more in the future to have less now (e.g., 90 dollars today rather than 100 dollars next month)—can be measured to assess the extremity with which future rewards are devalued. Some explorations have revealed that older adults are less likely than younger adults to discount future rewards (Li, Baldassi, Johnson, & Weber 2013; Löckenhoff, O’Donoghue, & Dunning, 2011; but see Sanchez-Roige et al., 2018). Given recent work that has demonstrated self-continuity as a driving factor in temporal discounting for young adults (Bartels & Urminsky, 2011; Ersner-Hershfield, Garton, et al., 2009), one might hypothesize that a similar mechanism is at work in an older population. While this has yet to be tested, Löckenhoff and Rutt (2017) do identify three studies that have directly explored the link between age and future self-continuity. Corroborating work focused on past self-continuity (Habermas & Köber, 2015; Sedikides et al., 2016), investigations probing the future have revealed a positive correlation between age and self-continuity (Hershfield, 2011; Rutt & Löckenhoff, 2016a, b). In particular, Rutt and Löckenhoff (2016b) charted the self-continuity associated with contemplations of multiple time points (from 3 months to 10 years from now) and found that after 1 year, older adults’ ratings of self-continuity begin to stabilize, whereas younger adults continued to experience a decrease in self-continuity with increased temporal distance.

Notwithstanding the critical importance of this work for development, decision-making, and self-construal, the temporal windows investigated to date have been relatively narrow given rapidly increasing life expectancies. For both older and younger adults, most of the reviewed literature has focused on “distant futures” that are between two and 10 years from now. Rising concerns about being able to financially support increased life spans have extended inquiries to include conceptualizations of oneself around retirement age. Investigations of imagining oneself 40 years in the future (Macrae et al., 2017) or seeing oneself through a virtual reality interface 50 years in the future (Hershfield et al., 2011) have revealed that a sense of present and future self-overlap (self-continuity) is a key determinant of prudent financial decisions (e.g., saving rather than spending). While these techniques may prove to be fruitful for improving decisions made for one’s future self in several domains, information about the components of mental imagery that characterize extraordinarily distant future simulations and the effects of age are currently missing from the literature (Löckenhoff & Rutt, 2017).

Extrapolating from the data at hand, one might presume that increasing temporal distance would be met with predictable changes in associated variables (e.g., abstraction, valence, and self-continuity). But what happens when the rules change and temporal distance is no longer met by the expected physical and mental decline currently associated with aging? And how might the simulations of older adults, who have less temporal distance to mentally traverse, compare to their younger counterparts when imagining an extremely distant future? It is precisely these issues—living longer without getting biologically older and the impact of highly relevant demographic variables—that are of interest to the present inquiry. In particular, the current work explores the mental construal of hypothetical aging scenarios and depicts current perceptions of life-span extension across different populations.

## The Current Research

Three experiments were designed to probe the characteristics associated with imagining an extraordinarily distant future under the presuppositions of regenerative aging interventions (procedures to halt or reverse the aging process). Studies 1 and 2 explored the characteristics of imagery associated with different counterfactual ages ranging from near the current species maximum (e.g., 120, 150) to more highly hypothetical ages (e.g., 201, 501). Study 3 probed the mental construal of extreme aging among different populations (life-extension supporters, students, Mechanical Turk workers) with a scenario that asked participants to imagine an average day in their life at 180 years old.

It is worth noting the hypotheticality of the scenario in which participants of the following empirical studies were asked to engage—imagining chronological without biological aging. One might wonder if it is even possible to imagine chronological without biological aging. While at its heart this is a deeply philosophical question, the psychology literature has demonstrated that it is not at all atypical for individuals to simulate counterfactual scenarios or situations well beyond current reality (Epstude & Roese, 2008; Suddendorf & Corballis, 1997). Thinking about the future, in particular, is believed to be even less restricted than thinking about the past (Van Boven, Kane, & McGraw, 2008). In short, prospection is a tool by which humans are able to simulate the unrealized, whether impossible or just slightly bizarre. Indeed, every time an individual engages in prospection (imagining the future), they are in fact imagining a hypothetical event.

Of course, mental simulations are heavily influenced by expectations about what the future will be like and common mental scripts—what we believe to be “typically” associated with aging (e.g., in a culture where getting married and having children in one’s 20’s is the norm, it is likely that young children will imagine their futures to include these activities). Simulating a life beyond one’s current life expectancy may then uncover insightful differences in underlying assumptions about what the future will be like or how one would feel after living for so many years. For those who have less experience with the idea of life extension, or find the notion of living longer less desirable, we expect to see the most notable differences when comparing simulations of an extremely distant future to simulations of the present (a control condition).

The key characteristics of mental imagery probed here include the following: valence, vividness, self-continuity, and visual perspective. The selection of these variables was informed by previous work that has investigated thinking about one’s future self within the boundaries of the current life expectancy (Hershfield et al., 2011; Macrae et al., 2017; Pronin et al., 2008; Pronin & Ross, 2006; Wakslak et al., 2008). All of these imagery qualities have been assessed using single-item, face valid measures adopted from the mental imagery literature (see Macrae et al., 2015; Tausen et al., 2018, 2019). Each of our three studies also considered perceptions of the likelihood and ethicality of life extension in order to accurately describe the samples (and their corresponding assumptions) under investigation in the current research. Study 3, in particular, assessed a unique population of individuals—life-extension supporters—who are already invested in the idea of living beyond the current life expectancy. As such, we expected this group of individuals to differ significantly from other populations not only in terms of how they imagine the distant future, but also their overarching beliefs about life extension.

Given the unprecedented and exploratory nature of this work, we did not have detailed hypotheses surrounding the specific characteristics of mental imagery that would (and would not) be impacted by imagining oneself beyond the current life expectancy under the guise of chronological without biological aging. The assessment of self-continuity is, however, the backbone of our investigation due to the weighty cognitive and behavioral implications of feeling disconnected from one’s future self. While previous research has suggested that distant future simulations are often characterized by a weaker sense of self-continuity than simulations of the near future, the mechanism underlying this effect (e.g., different mental or physical abilities, time in general, stereotypes about older adults, etc.) is not well understood. Thus, we remained open to the possibility that without the physical cues of aging, people could mentally transport themselves to an extremely distant future and still feel very connected to their future selves.

## Study 1: 120 Versus 150 Years Old

### Method

#### Participants/design

Power analyses estimated on a medium effect size (0.25) for three groups revealed that a sample size of 60 participants per group would result in 86% power. Preregistered sample size was set at 80 participants per condition.

Two hundred and forty participants were recruited online using Amazon’s Mechanical Turk (www.mturk.com). After reading a brief description of the task, Mechanical Turk workers who chose to take part were linked to a survey, which was generated and administered using Qualtrics (www.qualtrics.com). Mechanical Turk’s unique “worker id” numbers and IP addresses were recorded for each submitted survey, thus allowing multiple responses from the same individual to be excluded from data analysis. No responses came from repeated IP addresses. Eleven participants failed at least one of the manipulation checks and were dropped from the sample. Data analysis was performed on the remaining 229 participants (121 females, 1 nonbinary) aged between 18 and 77 (*M* = 36.66, *Mdn* = 33.00, *SD* = 12.74). The age distribution was non-normally distributed, with a skewness of 1.01 (*SE* = 0.16). Quartiles include the following age ranges: 18–28, 29–33, 34–43, and 44–77.

The study was reviewed and approved by Seattle Pacific University Institutional Review Board and preregistered on aspredicted.org. All participants were informed about the nature of the study prior to agreeing to participate and were able to withdraw at any point. The study employed a single factor (Imagery Condition: 120 years vs 150 years vs Control) between-participants design.

#### Materials/procedure

A short questionnaire was constructed to investigate the role of hypothetical aging on characteristics of mental imagery. After reading a brief description of the study participants were given the following information,

With the advancements of medicine and technology, our understanding of what it looks like to grow older is rapidly changing. Imagine that it is possible for you to grow older without experiencing any physical or mental decline by engaging in continuous regenerative interventions (procedures to halt or reverse the aging process). In other words, imagine it is possible to maintain optimal health, and fitness no matter how old you are—to spend more years alive without getting biologically older.

As an attention check, participants used a free response format to describe regenerative aging interventions in their own words. Participants were then randomly assigned to an imagery condition. Participants in the life-extension conditions were told:

Assume that you have just recently started regenerative interventions that will slow and possibly even reverse your aging process. On the next page, you will be asked to imagine what it would be like to be 120 (vs 150) years old and participating in a work-/job-related activity of your choice on a Monday morning.

Whereas participants in the control condition read,

Assume that you have just recently learned about regenerative aging interventions, but are currently not eligible to take part. On the next page, you will be asked to imagine being your current age and participating in a work-/ job-related activity of your choice on a Monday morning.

Participants in all conditions were then asked to report what activity they would be imagining and the year associated with the age they were asked to imagine—questions that would serve as our final two manipulation checks. On the next page, participants were given 30 s to imagine the scenario.

Following the task, participants were asked to describe their mental imagery (free response format) and respond to a number of questions probing the characteristics of their mental imagery. Specifically, participants were asked to report on the valence (very negative/very positive) and vividness of their imagery (not very vivid/very vivid) as well as how engaged they imagined being in their work (not at all/very much) and self-continuity—the extent to which they felt the person they were imagining was actually them (not at all like me—it felt like I was imagining a different person/very much like me—it felt like I was imagining myself) on 100-point analog scales with the aforementioned anchors. Similarity between their current job/work activities and the ones they imagined was also probed using a 4-point Likert scale. Participants also used forced choice questions to report the visual perspective that characterized their imagery (i.e., first person vs third person vs both perspectives represented equally). After completing the core dependent variable questions, participants were asked to report on the likelihood that regenerative aging techniques will be available and effective in the future and whether they find engaging in aging regeneration to be morally/ethically acceptable on 100-point analog scales with appropriate anchors. Finally, participants answered demographic questions including their age and how religious/spiritual they consider themselves, were thanked, and debriefed.

### Results

#### Imagery characteristics

A multivariate analysis of variance (MANOVA) revealed an effect of Imagery Condition on the sense of self-continuity [*F*(2, 224) = 3.92, *p* = .021, *η*_*p*_^2^ = .034] and job consistency [*F*(2, 224) = 9.77, *p* < .001, *η*_*p*_^2^ = .08], which characterized mental imagery. No other effects of aging condition were found on any of the other dependent variables (valence, vividness, and job engagement) that were collected (*F’s* < 1.02). It is worth noting that the MANOVA approach was utilized here because individualized ANOVAs do not have the ability to account for shared variance (e.g., they ignore that there is likely a relationship between the dependent variables because they are coming from the same people—for example, those naturally higher in imagery ability may show both higher vividness and self-continuity scores, or those who imagine engaging in the same job that they are doing now may also report a stronger connection to their future self). Follow-up analyses using individual ANOVAs demonstrated an identical pattern of results.

**Figure 1.**
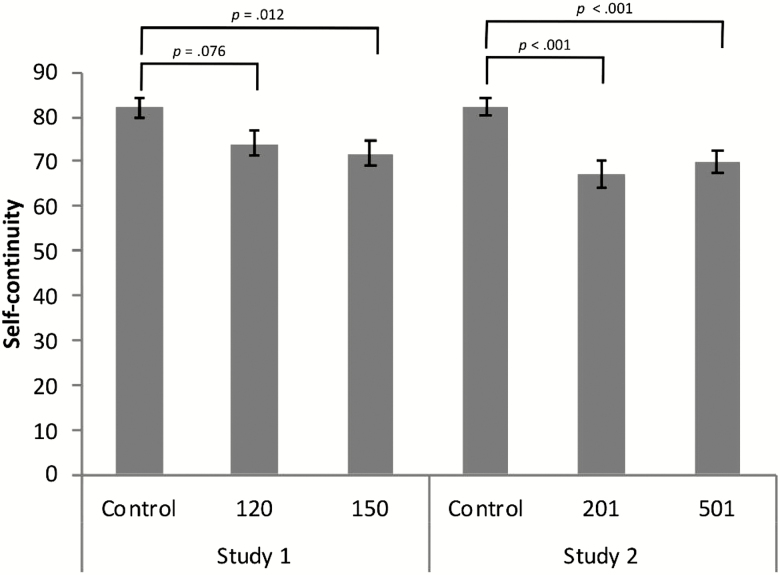
Judgments of self-continuity associated with imagining a typical workday in the present compared to 120, 150 (Study 1) 201, and 501 years old (Study 2). Errors bars represent ± 1 SEM. All measures were assessed using a 100-point analog scale.

Post-hoc pairwise comparisons (Bonferroni corrected) revealed that imagining a work activity at one’s current age was characterized by a marginally greater sense of self-continuity (*M* = 82.07, *SD* = 20.39) than imagining being 120 years old [*M* = 74.14, *SD* = 25.97, *t*(155) = 2.10, *p* = .076, *d* = .39, 95% confidence interval (CI): 0.48, 15.37] and a significantly greater sense of self-continuity than imagining being 150 years old [*M* = 71.78, *SD* = 23.78, *t*(143) = 2.80, *p* = .012, *d* = .47, 95% CI: 3.02, 17.56; Figure 1]. Job consistency also differed, such that imagining one’s current age was characterized by a job more similar to the present one [*M* = 3.35, *SD* = 0.89) than a job that one imagined having at 120 years old [*M* = 2.79, SD = 1.12, *t*(153.18) = 3.49, *p* = .002, *d* = .55, 95% CI: 0.24, 0.88] and 150 years old [*M* = 2.62, *SD* = 1.07, *t*(135.64) = 4.41, *p* < .001, *d* = .75, 95% CI: 0.40, 1.05]. No significant differences in self-continuity or job consistency between the 120-year-old and 150-year-old conditions were found (*t*’s < .94). A χ ^2^ test revealed that the use of first person, third person, and a combination of the two perspective was not significantly differentially distributed across conditions, χ ^2^ (4, *N* = 229) = 7.99, *p* = .092, such that 58.90% of current age simulations were characterized by an exclusively first-person perspective, compared to 54.76% of 120-year-old simulations and 50.0% of 150-year-old simulations.

#### Age as a covariate

Analysis of covariance (ANCOVA), controlling for age, was conducted for each of the imagery characteristics (vividness, valence, self-continuity, and job consistency). These analyses revealed an identical pattern of results as the MANOVA reported above, with significant effects of Imagery Condition on self-continuity and job overlap, but no other significant effects. These analyses also revealed a significant effect of age on valence, *F*(1, 222) = 5.56, *p* = .019, *η*_p_^2^ = .24, and self-continuity, *F*(1, 222) = 6.97, *p* = .009, *η*_p_^2^ = .03, such that as participants’ age increased, they reported more positive simulations and a greater sense of self-continuity. No other significant effects of the age were found.

#### Likelihood and ethicality

One-sample *t*-tests compared participants’ ratings of the likelihood that life-extension techniques will be common and effective and the ethicality of engaging with relevant interventions to the midpoint of the scale. Results revealed that participants’ ratings of the likelihood of life-extension techniques (*M* = 52.47, *SD* = 28.33) did not significantly differ from the midpoint of the scale [*t*(228) = 1.32, *p* = .19, 95% CI: −1.22, 6.16], but participants’ ratings of how ethical these interventions are (*M* = 61.59, *SD* = 29.69) did significantly differ from the midpoint [*t*(228) = 5.91, *p* < .001, 95% CI: 7.72, 15.45], such that participants’ responses trended toward an ethical assessment of these interventions (Table 1).

**Table 1.** Descriptive Statistics Depicting Average Ratings Pertaining to the Perceived Likelihood and Ethicality of Life-Extension Techniques and Inferential Statistics Depicting Results of One-Sample *t*-Tests Comparing Mean Values to Midpoint (50) of the Scale

	Likelihood			Ethicality		
	*M* (*SD*)	*t*-value	95% CI_diff_	*M* (*SD*)	*t*-value	95% CI_diff_
Study 1 (*N* = 228)	52.47 (28.33)	1.32	−1.22, 6.16	61.59 (29.69)	5.91**	7.72, 15.45
Study 2 (*N* = 271)	50.08 (29.74)	0.045	−3.48, 3.64	62.33 (27.58)	7.36**	9.03, 15.63
Study 3: Students (*N* = 86)	40.49 (27.04)	−3.26*	−15.31, −3.71	40.09 (26.07)	−3.52**	−15.50, −4.32
Study 3: Mechanical Turk Workers (*N* = 78)	44.53 (28.57)	−1.69	−11.92, 0.97	57.36 (30.17)	2.15*	0.56, 14.16
Study 3: Life-Extension Supporters (*N* = 38)	89.84 (15.51)	15.84**	34.75, 44.94	99.03 (2.81)	107.4**	48.10, 49.95

*Note*: Values denoted with an asterisk were significantly different from the midpoint of the scale: * *p* < .05; ** *p* < .001. CI = confidence interval.

#### Exploratory correlations

Vividness positively correlated with all measures except age and religiosity, such that increased vividness was associated with increased valence [*r*(229) = .48, *p* < .001], self-continuity [*r*(229) = .51, *p* < .001], job consistency (*r*(227) = .16, *p* = .017), work engagement [*r*(229) = .41, *p* < .000], likelihood [*r*(229) = .26, *p* < .001], and ethicality [*r*(229) = .17, *p* = .011]. Valence positively correlated with self-continuity [*r*(229) = .46, *p* < .001], work engagement [*r*(229) = .66, *p* < .001], likelihood [*r*(229) = .18, *p* = .006], age [*r*(228) = .15, *p* = .022], and religiosity [*r*(229) = .13, *p* = .043]. Self-continuity positively correlated with job consistency [*r*(227) = .19, *p* = .004], job engagement [*r*(229) = .47, *p* < .001], likelihood [*r*(229) = .29, *p* < .001], ethicality [*r*(229) = .29, *p* < .001], and age [*r*(228) = .16, *p* = .015]. Job consistency positively correlated with ethicality [*r*(227) = .16, *p* = .016]. Job engagement positively correlated with likelihood [*r*(229) = .27, *p* < .001], ethicality [*r*(229) = .18, *p* = .006], and religiosity [*r*(229) = .15, *p* = .02]. Likelihood positively correlated with ethicality [*r*(229) = .50, *p* < .001]. Ethicality positively correlated with age [*r*(228) = .17, *p* = .011; Table 2].

**Table 2.** Exploratory Correlations Between All Continuous Measures Collected in Study 1

	1	2	3	4	5	6	7	8	9
1. Vividness	—								
2. Valence	.48**	—							
3. Self-continuity	.51**	.46**	—						
4. Job consistency	.16*	.02	.19**	—					
5. Job engagement	.41**	.66**	.47**	.05	—				
6. Likelihood	.26**	.18**	.29**	.13	.27**	—			
7. Ethicality	.17*	.12	.29**	.16*	.18**	.50**	—		
8. Age	.11	.15*	.16*	−.03	.12	.02	.17*	—	
9. Religiosity	.08	.13*	.07	−.01	.15*	.03	−.1	.12	—

*Note*: Values denoted with an asterisk were significantly correlated: * *p* < .05; ** *p* < .001.

### Discussion

Study 1 explored the mental characteristics associated with imagining a hypothetical aging scenario. Mental simulations of the present and extremely distant future did not differ in standard imagery components (i.e., valence, vividness, and visual perspective), but were distinct in the extent to which individuals felt a sense of self-continuity and the overlap between their current and imagined job. Furthermore, 30 years of temporal distance between the two hypothetical aging conditions did not significantly impact characteristics of the mental simulation such as self-continuity, vividness, and valence. What this suggests is that temporal distance may only impact perceived self-continuity if biological aging is involved. If this is the case, then differences between the control and the chronological without biological aging conditions might be better attributed to differences in hypotheticality rather than temporal distance.

Examining age as a covariate revealed an identical pattern of results, but also identified age as a significant predictor of self-continuity, such that across all conditions age was positively correlated with a sense of connectedness to one’s future self. Exploratory correlations corroborated this finding and also revealed a significant correlation between age and the valence of the mental simulation, such that the older participants were the more positively they rated their mental imagery. Extending the current investigation, Study 2 sought to push the boundaries of life extension even further by investigating simulations of being 201 and 501 years old—significantly more hypothetical time points that are well beyond the maximum life expectancy for humans and spaced 300 (vs 30) years apart.

## Study 2: 201 Versus 501 Years Old

### Participants/Design

Power analyses estimated on a medium effect size (.25) for three groups revealed that a sample size of 60 participants per group would result in 86% power. Preregistered sample size was set at 80 participants per condition.

Two hundred and eighty-four participants were recruited online using Amazon’s Mechanical Turk (www.mturk.com). After reading a brief description of the task, Mechanical Turk workers who chose to take part were linked to a survey, which was generated and administered using Qualtrics (www.qualtrics.com). Mechanical Turk’s unique “worker id” numbers and IP addresses were recorded for each submitted survey, thus allowing multiple responses from the same individual to be excluded from data analysis. No responses came from repeated IP addresses. Thirteen participants failed at least one of the manipulation checks and were dropped from the sample. Data analysis was performed on the remaining 271 participants (137 female) aged between 20 and 67 (*M* = 35.55, *Mdn* = 33.00, *SD* = 11.14). One participant reported their age as 5, but passed all manipulation checks. Thus, the participant’s age is not included in the measures of central tendency reported here, but their data are included in the remaining data analyses. The age distribution was nonnormally distributed, with a skewness of .96 (*SE* = 0.15). Quartiles include the following age ranges: 20–27, 28–33, 34–41, and 42–67.

The study was reviewed and approved by the Seattle Pacific University Institutional Review Board and preregistered on aspredicted.org. All participants were informed about the nature of the study prior to agreeing to participate and were able to withdraw at any point. The study employed a single factor (Imagery Condition: 201 years old vs 501 years old vs Control) between-participants design.

### Materials/Procedure

Procedures were identical to that of Study 1, with the exception that in the two experimental conditions participants were asked to imagine being either 201 or 501 years old while performing a work activity. All other measurements and procedures remained the same.

### Results

#### Imagery characteristics

MANOVA revealed an effect of Imagery Condition on the sense of self-continuity [*F*(2, 268) = 10.04, *p* < .001, *η*_*p*_^2^ = .07], and job consistency [*F*(2, 268) = 17.27, *p* < .001, *η*_p_^*2*^ = .114], which characterized mental imagery. No other effects of aging were found (*F’s* < 1.2). As in Study 1, the MANOVA approach was utilized to account for shared variance in the dependent variables. Follow-up analyses using individual ANOVAs demonstrated an identical pattern of results.

Post-hoc pairwise comparisons (Bonferroni corrected) revealed that imagining a work activity at one’s current age was characterized by a greater sense of self-continuity (*M* = 82.11, *SD* = 17.91) than imagining being 201 years old [*M* = 67.17, *SD* = 26.89, *t*(147.12) = 4.32, *p* < .001, *d* = .66, 95% CI: 8.10, 21.78] and 501 years old [*M* = 70.14, *SD* = 25.06, *t*(170.40) = 3.75, *p* < .001, *d* = .55, 95% CI: 5.68, 18.27; Figure 1]. Job consistency also differed, such that imagining one’s current age was characterized by a job more similar to the present one (*M* = 3.36, *SD* = 0.99) than a job that one imagined having at 201 years old [*M* = 2.48, SD = 1.12, *t*(168.81) = 5.50, *p* < .001, *d* = .84, 95% CI: 0.56, 1.19] and 501 years old [*M* = 2.58, *SD* = 1.16, *t*(180.83) = 4.90, *p* < .001, *d* = .73, 95% CI: 0.46, 1.09]. No significant differences in self-continuity or job consistency between the 201-year-old and 501-year-old conditions were found. A χ ^2^ test revealed that the use of first person, third person, and a combination of the two perspective was differentially distributed across conditions, χ ^2^ (4, *N* = 271) = 9.79, *p* = .044, such that 60% of current age simulations were characterized by an exclusively first-person perspective, compared to 39.5% of 201-year-old simulations and 47.4% of 501-year-old simulations.

#### Age as a covariate

ANCOVA controlling for age were conducted for each of the imagery characteristics (vividness, valence, self-continuity, and job consistency). These analyses revealed an identical pattern of results as the MANOVA reported above, with significant effects of condition on self-continuity and job overlap, but no other significant effects. Analyses also revealed a significant effect of the age, on vividness [*F*(1,267) = 7.94, *p* = .005, *η*_p_^2^ = .03], valence [*F*(1,267) = 5.04, *p* = .026, *η*_p_^2^ = .02], and self-continuity [*F*(1,267) = 12.40, *p* = .001, *η*_p_^2^ =. 04]. No other significant effects of age were found.

#### Likelihood and ethicality

One-sample *t*-tests compared participants’ ratings of the likelihood that life-extension techniques will be common and effective and the ethicality of engaging with relevant interventions to the midpoint of the scale. Results revealed that participants’ ratings of the likelihood of life-extension techniques (*M* = 50.08, *SD* = 29.74) did not significantly differ from the midpoint of the scale [*t*(270) = .045, *p* = .96, 95% CI: −3.48, 3.64], but participants’ ratings of how ethical these interventions are (*M* = 62.33, *SD* = 27.58) did significantly differ from the midpoint [*t*(270) = 7.36, *p* < .001, 95% CI: 9.03, 15.63], such that participants responses trended toward an ethical assessment of these interventions (Table 1).

#### Exploratory correlations

Vividness positively correlated with all other measures except job consistency and ethicality, such that increased vividness was associated with increased valence [*r*(271) = .35, *p* < .001], self-continuity [*r*(271) = .50, *p* < .001], likelihood [*r*(271) = .17, *p* = .005), work engagement [*r*(271) = .44, *p* < .000], religiosity [*r*(271) = .12, *p* = .041], and age [*r*(271) = .17, *p* = .004]. Valence positively correlated with self-continuity [*r*(271) = .42, *p* < .001], work engagement [*r*(271) = .63, *p* < .001], job consistency [*r*(271) = .18, *p* = .003], ethicality [*r*(271) = .21, *p* = .001], and age [*r*(271) = .13, *p* = .031]. Self-continuity positively correlated with work engagement [*r*(271) = .35, *p* < .001], likelihood [*r*(271) = .26, *p* < .001], ethicality [*r*(271) = .16, *p* = .009], and age [*r*(271) = .22, *p* < .001]. Job consistency positively correlated with engagement [*r*(271) = .16, *p* = .01]. Job engagement positively correlated with likelihood [*r*(271) = .14, *p* = .024] and ethicality [*r*(271) = .16, *p* = .011]. Likelihood positively correlated with ethicality [*r*(271) = .42, *p* < .001]. Ethicality negatively correlated with religiosity [*r*(271) = −.19, *p* = .002]. Age and religiosity showed a marginally significant positive correlation [*r*(271) = .11, *p* = .068; Table 3].

**Table 3.** Exploratory Correlations Between All Continuous Measures from Study 2

	1	2	3	4	5	6	7	8	9
1. Vividness	—								
2. Valence	.35**	—							
3. Self-continuity	.50**	.42**	—						
4. Job consistency	.05	−.18**	.09	—					
5. Job engagement	.44**	.63**	.35**	−.15*	—				
6. Likelihood	.17**	.11	.26**	.04	.14*	—			
7. Ethicality	.02	.21**	.16**	−.06	.15*	.42**	—		
8. Age	.18**	.13*	.22**	.06	.1	−.04	.09	—	
9. Religiosity	.13*	−.06	.06	.05	−.01	−.04	−.19**	.1	—

*Note*: Values denoted with an asterisk were significantly correlated: * *p* < .05; ** *p* < .001.

### Discussion

Study 2 replicated and extended the findings from Study 1 by exploring more extreme (and thus more hypothetical) temporal distances. Both years from the present and years between hypothetical future ages were exaggerated such that participants in experimental conditions simulated being 201 or 501 years of age. Corroborating Study 1, the results revealed a reduced sense of self-continuity and job consistency in the hypothetical aging (vs control) conditions. The use of first- versus third-person visual perspective (or a combination of the two), however, was differentially distributed across the three conditions, such that present simulations were more likely than distant future simulations to be imagined from a first-person perspective. These findings departed from those in Study 1, where shifts in visual perspective failed to reach significance. The pattern of results in the two studies, however, is consistent as Study 1 demonstrated fewer reports of exclusively first-person simulations with increased imaginary age. Controlling for age in the analyses revealed an identical pattern of results and substantiated the claim that age was a significant predictor of self-continuity (even when imagining 201+ years into the future). Age also significantly correlated with valence such that older participants reported more positive imagery, as was the case in Study 1.

Replicating and extending the results from Study 1, Study 2 demonstrated that exaggerated temporal distance (300 years) does not appear to affect the construal (e.g., vividness, valence, or self-continuity) of mental simulations characterized by hypothetical aging. Whether participants imagined themselves being 201 or 501 years old did not significantly impact the characteristics of their mental simulations, further supporting the notion that the attenuation of a sense of connectedness to one’s future self may be tied to the mental or physical decline associated with aging.

The final study explored perceptions of hypothetical aging among different populations (i.e., students, Mechanical Turk workers, and life-extension supporters) and probed the characteristics of mentally simulating life beyond the life span for members in each of these groups. The three groups tested here were chosen in order to represent individuals with varying levels of knowledge about life-extension techniques. In addition, these three groups vary in age and religiosity and are thus able to provide a more holistic view on perceptions of life extension.

## Study 3: Different Populations Imagine Being 180 Years Old

### Method

#### Participants/Design

Two-hundred and twenty participants were recruited from one of three platforms: Amazon’s Mechanical Turk (*N* = 85), Seattle Pacific University’s participant pool (*N* = 94), and a community of life-extension supporters (*N* = 41). After reading a brief description of the task, those who chose to take part were linked to a survey, which was generated and administered using Qualtrics (www.qualtrics.com). Eighteen participants failed at least one of the manipulation checks and were dropped from the sample. Data analysis was performed on the remaining 202 participants (116 female, 1 nonbinary) aged between 18 and 78 (*M* = 32.17, *Mdn* = 27.5, *SD* = 14.88). The age distribution was nonnormally distributed, with a skewness of 1.11 (*SE* = 0.16). Quartiles include the following age ranges: 18–19, 20–27, 28–39, and 40–78.

The study was reviewed and approved by Seattle Pacific University Institutional Review Board. All participants were informed about the nature of the study prior to agreeing to participate and were able to withdraw at any point. The study employed a single factor (Group: Mechanical Turk workers, students, life-extension supporters) between-participants design. Life-extension supporters were recruited through their registration and attendance at the Undoing Aging conference held in Berlin, Germany, on March 15–17, 2018. At the end of an unrelated presentation by the second author, participants were invited to take part in a “short questionnaire about robust life extension.” No incentives were offered for participation.

#### Materials/Procedure

All procedures and materials were identical to the previous studies with the exception that participants were asked to imagine “an average day in your life when you are 180 years old.” Participants were not asked about job consistency (because they did not necessarily imagine a work scenario). An additional question was added to the survey, which asked participants to communicate (via a free response format) how long they would like to live if it was possible for them to live longer without experiencing the physical decline of aging.

### Results

#### Imagery characteristics

MANOVA revealed a significant main effect of Group on the valence [*F*(2, 199) = 9.76, *p* < .001, *η *_p_^2^ = .09] and self-continuity [*F*(2, 199) = 14.07, *p* < .001, η _p_^2^ = .12] and a marginally significant effect of condition on the vividness of mental imagery [*F*(2, 199) = 2.85, *p* = .06, *η* _p_^2^ = .028]. As in Studies 1 and 2, the MANOVA approach was utilized to account for shared variance in the dependent variables. Follow-up analyses using individual ANOVAs demonstrated an identical pattern of results.

**Figure 2.**
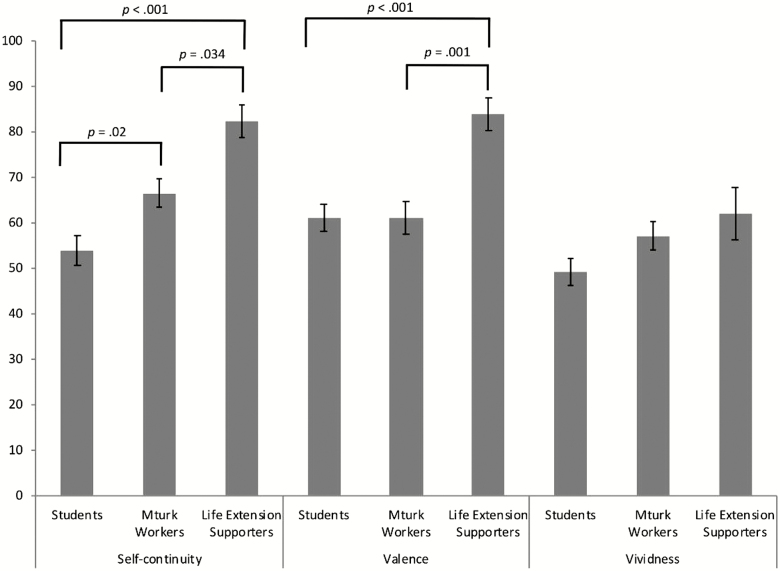
Judgments of valence, vividness, and self-continuity associated with imagining being 180 years old across each of the three distinct groups (students, Mechanical Turk workers, life-extension supporters). Errors bars represent ± 1 SEM. All measures were assessed using a 100-point analog scale.

Post-hoc (Bonferroni corrected) pairwise comparisons revealed no significant differences between any of the three groups in vividness. Life-extension supporters reported significantly more positive imagery than both Mechanical Turk workers (*p* = .001, 95% CI: 8.03, 36.46) and the student sample (*p* < .001, 95% CI: 8.35, 36.42). Mechanical Turk workers did not significantly differ from students (95% CI: −10.81, 11.10) on valence ratings. Life-extension supporters reported that their simulations were characterized by significantly more self-continuity than did Mechanical Turk workers (*p* = .034, 95% CI: 0.80, 28.24) and students (*p* < .001, 95% CI: 12.96, 40.06). MTurk workers also significantly differed from the student sample, reporting higher levels of self-continuity (*p* = .02, 95% CI: 1.41, 22.56; Figure 2).

Chi-square analysis revealed that the distribution of visual perspectives was different across groups [χ ^2^(4, *N* = 202) = 11.20, *p* = .02]. Students reported the majority (39.5%) of their mental simulations to be from a third-person perspective, whereas 26.7% of simulations were characterized by both first- and third-person perspective and 33.7% of simulations were characterized by first-person imagery. The patterns of first- and third-person imagery were reversed for the other two groups. Mechanical Turk Workers reported the majority of their mental simulations to be characterized by first-person imagery (46.15%), whereas 29.49% were characterized by both first- and third-person imagery, and the remaining 24.36% were characterized by third-person imagery. Life-extension supporters reported the majority of their mental simulations to be characterized by first-person imagery (57.90%), whereas 29.00% were characterized by both first- and third-person imagery, and the remaining 13.16% were characterized by third-person imagery.

#### Age as a covariate

Although the three samples significantly differed in age, with students being the youngest (*M* = 20.17, *SD* = 14.88), followed by Mechanical Turk workers (*M* = 37.86, *SD* = 11.01) and the life-extension supporters (*M* = 48.00, *SD* = 16.14), controlling for age in our analyses resulted in an identical pattern of results for all mental imagery characteristics, suggesting that the variance in dependent variables cannot be explained by age differences between the three groups. Analyses revealed no significant effect of age on any of the imagery variables.

#### Likelihood, ethicality, and desired length of extended life

MANOVA revealed a significant effect of Group on the perceived likelihood [*F*(2, 195) = 48.11, *p* < .010, η _p_^2^ = .33] and ethicality [*F*(2, 195) = 66.24, *p* < .001, *η* _*p*_^2^ = .41] of life-extension techniques. Post-hoc pairwise comparisons (Bonferroni corrected) revealed that the life-extension supporters reported life extension to be significantly more likely (*M* = 89.84, *SD* = 3.62) than students (*M* = 40.49, *SD* = 2.92, *p* < .001, 95% CI: 36.72, 62.06) and the Mechanical Turk workers (*M* = 44.53, *SD* = 3.23, *p* < .001, 95% CI: 32.64, 58.31). The student and Mechanical Turk group did not significantly differ on the likelihood measure (*p* = 1, 95% CI: −5.98, 13.81). Life-extension supporters also rated life extension to be significantly more ethical (*M* = 99.03, *SD* = .46) than students (*M* = 40.09, *SD* = 2.81, *p* < .001, 95% CI: 46.73, 71.54) and Mechanical Turk workers (*M* = 57.36, *SD* = 3.42, *p* < .001, 95% CI: 29.02, 54.15) on ratings of ethicality. Students and Mechanical Turk workers also differed on ratings of ethicality (*p* < .001, 95% CI: 7.86, 27.23;Table 1).

Finally, participants were asked how long they would like to live if they could do so without getting biologically older. Of the 78 Mechanical Turk participants who answered this question, 3.8% said they did not know, 3.8% said they would like to live indefinitely/forever, and 2.6% qualified that their preference would depend on social factors (e.g., the relationships they had), 89.7% provided a numerical response (*M* = 16,727.81, *Mdn* = 137.5, *SD* = 11,986.65). Of the 86 students, 2.3% left the question blank, 3.5% suggested they would like to live forever, 3.5% clarified they would never take part in relevant interventions, 8.1% said they would like to live until they achieved a particular purpose or meaning in life, 4.7% qualified that their preference would depend on social factors (e.g., the relationships they had/ people they loved were alive), and 77.9% provided a numerical value (*M* = 124.06, *Mdn* = 100, *SD* = 65.61). Of the 38 life-extension supporters who answered the question, 2.6% responded they did not know, 57.9% said they would like to life indefinitely/forever, 10.5% qualified that they would like to live as long as physically able or until they died via a means not related to aging, 2.6% suggested they would like to live as long as they experienced purpose, and 13.2% responded with a numerical value (*M* = 1,044.00, *Mdn* = 200, *SD* = 1,672.55).

### Discussion

This final study, comparing mental characteristics of hypothetical aging scenarios across different groups, suggests that there are a multitude of factors that shape the mental construction of a hypothetical future event. Across the board, life-extension supporters reported more positive simulations characterized by a greater sense of self-continuity than any other group. Not only was the average rating of self-continuity for this group comparable to control conditions (imagining the present) in previous studies, but life-extension supporters were the only group to report their mental simulations took a predominately first-person perspective. Perhaps less surprisingly, the life-extension group also significantly differed in their ratings of the ethicality and likelihood of life-extension technologies. Exploratory analyses confirmed that across all three groups ethicality and likelihood were both significantly correlated with self-continuity. What this suggests is that buy-in and support for “undoing aging” may enhance self-continuity when mentally transcending temporal distance. Of course, future empirical work will be necessary to test this conjecture.

## General Discussion

Three experiments explored the characteristics associated with imagining life beyond the current life span under presuppositions of suspended biological aging. Whether asked to imagine being 120, 150, 180, 201, or 501 years old, the results demonstrated that participants were able to imagine hypothetical future scenarios as vividly as simulations of the present. Critically, however, imagining oneself beyond the current life expectancy was characterized by a reduction in self-continuity (compared to imagining the present). Outside of these components, the psychological laws of temporal distance seem to be suspended in the extremely distant future. When comparing the experimental (hypothetical aging) conditions, temporal distance (e.g., the 30 years between 120 and 150 or the 300 years between 201 and 501) did not significantly alter characteristics (e.g., vividness, valence, and self-continuity) of mental simulations. What this suggests is that biological aging itself may be a key mechanism driving the decrease in self-continuity that is often associated with imagining oneself in the distant future. If this is the case, then differences in self-continuity ratings between the control and experimental conditions may be better attributed to the hypotheticality component of psychological distance rather than the temporal components. Future work will be necessary to unpack the relationship between different aspects of psychological distance on the effects of self-continuity (see Tausen et al., 2019 for discussion).

### Individual Differences

Demographic variables were probed here as they related to the components of mentally simulating life beyond the life span. Students, Mechanical Turk workers, and life-extension supporters varied widely in the characteristics of their mental simulations. Most notably, the life-extension supporters showed a greater sense of self-continuity and positivity when imagining themselves in the extremely distant future. The relationship between support for life extension and valence was also evidenced in exploratory correlational analyses, such that the more feasible life extension was perceived to be, the more positive (Study 1) and ethical (Studies 1 and 2) it was rated. With the exception of the life-extension group, participants did not perceive aging interventions to be particularly likely. Group assessments of how ethical it would be to engage in these types of life-extending interventions did lean toward moral acceptance. However, the student sample assessed here, which was also particularly high in religiosity, diverged significantly from the other two groups—rating life-extension techniques to be considerably less ethical (and less vivid). A relationship between religiosity and ethicality was also seen in Study 2, such that the more religious participants claimed to be, the less ethical they perceived regenerative aging techniques. These findings reinforce the work of Ballinger and colleagues (2017), who identified that religiosity was inversely correlated with the desirability of increasing one’s own life beyond the expected life span. More generally, Lang and Rupprecht (2019) have identified unique mindsets that shape individual cognitions around longevity across the life span. The current findings emphasize the importance of assessing individual differences associated with beliefs about life-extension techniques and demonstrate that the perceived likelihood and ethicality shape the mental landscape of life beyond the current life expectancy.

The age range of our samples also varied widely, allowing us to explore potential relationships between current age and the characteristics of mentally simulating extreme aging. Given the critical role of temporal distance on construal of the self and future events (Ersner-Hershfield, Garton, et al., 2009; Liberman & Trope, 1998), one might expect older adults, with correspondingly shorter temporal distances to the imagined time point, to exhibit a more concrete and connected future mental simulation. Effects of age as a covariate in our model do support this hypothesis. In Studies 1 and 2, age was identified as a significant predictor of the valence and self-continuity that characterized a mental simulation. Exploratory correlations revealed the nature of these effects, such that the older participants were, the more positively they rated their imagery and also the more connected they felt to their extremely distant future selves. These findings were independent of condition, such that age did not differentially impact, for instance, simulations of 120 years old relative to simulations of 150 years old. Rather, age was more generally associated with positivity and a sense of connection to one’s future self. These trends are consistent with previous work identifying that age was positively correlated with a “Utopian Vision” of life extension (Kogan et al., 2011), as well as the more general tendency for older adults to focus on positive (vs negative) information (Scheibe & Carsensen, 2010) and report higher levels of self-continuity than younger adults when imagining the future. Beyond valence, the current work is the first to suggest that age is positively correlated with the self-continuity that characterizes imagining oneself living well beyond the current life expectancy.

A relationship between age and the reported vividness of a mental simulation was also seen in Study 2, such that the older participants were, the more vivid their mental simulations of the extremely distant future (e.g., 201/501). While previous work has found an opposite trend, a reduction in vividness and episodic specificity with increased age (Addis et al., 2008), we cannot rule out the possibility that suspended biological aging scenarios are constructed differently than simulating more realistic self-selected time points in one’s own future. One potential explanation might lie in the type of detail that populated extremely distant future simulations. The same body of work that demonstrated a reduction in episodic specificity for older adults also identified that older adults incorporate more external detail into their future simulations (Addis et al., 2008). To the extent that external detail maps onto reported vividness of hypothetical aging scenarios, it is possible that with increased years comes a paradoxically richer representation of the extremely distant future. However, caution should be taken when inferring any relationship as the correlation between age and vividness was not evidenced in Study 1. Future work will be necessary to examine the reliability of this finding.

### Self-Continuity

With advancements in medicine and technology, there is a growing interest in the idea of life extension. The enthusiasm for halting biological aging is palpable among the community of life-extension supporters, yet the current results provide a more generalizable assessment of the perceptions of these ideas across a wide variety of participants (see also Partridge, Lucke, Bartlett, & Hall, 2009). While not necessarily morally opposed, people of variable demographics seem to be generally skeptical of the feasibility of such life-altering advancements (see also Pew Research Center 2013a,b & 2014). Of practical and theoretical consequence, our results demonstrate that imagining the extremely distant future, even when characterized by the suspension of biological aging, is associated with a sense of separation between one’s current and distant future self. This effect was seen both in the perceived overlap between one’s real and imagined self as well as one’s present and future activities (i.e., the job one would be performing) and remained significant after controlling for age. Corroborating and extending previous work looking at typical aging (Rutt & Löckenhoff, 2016b), chronological aging characterized by suspended biological aging is also associated with decreased self-continuity. Compounding environmental and ethical concerns about life extension (Schneider, 2009), increased years to live may come at the psychological cost of feeling disconnected from one’s future self.

It is well established that individuals often fail to delay gratification in the present to their own (future) detriment (Bartels & Rips, 2010; Ersner-Hershfield, Garton, et al., 2009; Ersner-Hershfield, Wimmer, et al., 2009). How much more, then, might this future self-neglect be problematic should one live well beyond 100 years of age? Without a heightened sense of self-continuity, it is likely that extended life spans would merely compound the problems of immediate gratification and future neglect currently experienced when it comes to both the self (physical and financial) and the environment. Interestingly, some evidence suggests that older adults tend to be less prone to temporal discounting, particularly when considering gains in the future (Löckenhoff, O’Donoghue, & Dunning, 2011). While these investigations have not been directly linked to self-continuity, the potential mediating role of a sense of connectedness to one’s future self is an important and underexplored topic that might help to explain some of the cognitive and behavioral differences between older and younger adults when thinking about the future (also see Löckenhoff et al., 2011). Despite these well-established differences, explorations have been limited to relatively short time periods. Although age was positively correlated with self-continuity in the present research, extremely distant future simulations were uniformly characterized by a reduction in self-continuity. What this suggests is that the heightened future self-continuity experienced by older adults is unlikely to translate into potential future benefits should the possibility to live well beyond the current life span become a reality.

### Practical and Theoretical Considerations

Bearing in mind the trends for increased life expectancies across the globe, great consideration should be taken to further develop techniques, such as directed first-person simulations and immersive technologies (for individuals of all ages), that can be employed to increase the sense of self-continuity that accompanies imagining one’s distant future self (Hershfield et al., 2011; Macrae et al., 2017; Pronin et al., 2008; Pronin & Ross, 2006; Wakslak et al., 2008). Enhancing self-continuity via these means may very well help individuals circumvent problems associated with failing to plan for the distant future. Another potential remedy (at least for those who are not ethically opposed) may lie simply in the increased understanding of, and advancements in, robust life-extension techniques. Consistent with the predictions from Construal Level Theory about the consequences of increased hypotheticality (Trope & Liberman, 2010), the current studies demonstrated that ratings of likelihood were highly correlated with a number of the basic characteristics of mentally simulating an extremely distant future. Of particular consequence, the perceived likelihood of viable life-extension techniques corresponded to how much overlap one experienced between their imagined and distant future selves. If extreme life extension is perceived as realistic, it may be possible to mentally traverse extraordinary temporal distance while still feeling deeply connected to one’s distant future self. This was true for life-extension supporters whose average ratings of self-continuity when simulating oneself at 180 years old looked much like participants’ ratings in control conditions (imagining the present) in previous studies.

Moreover, hypothetical future aging scenarios that varied in temporal distance did not differ in self-continuity. Woven into the framework of Construal Level Theory (Trope & Liberman, 2010), it is possible that decreased self-continuity is a natural consequence of psychological distance, but that perceived hypotheticality, even more than temporal distance, is a key determinant (see Tausen et al., 2019). Future work will be necessary to test these hypotheses and disentangle the roles of hypotheticality and temporal distance when investigating the sense of self-continuity that accompanies prospection. While there are undoubtedly countless approaches to such questions, leveraging scenarios denoted by chronological, without corresponding biological, aging may provide one avenue by which novel insights about psychological distance and Construal Level Theory could be obtained.

Beyond a sense of present and future self-overlap, a concern highlighted by a number of the participants sampled here (when asked how long they would like to live) is that of lacking social connection and purpose during the extended years in life. With the exception of the life-extension supporters, many participants’ responses about the desired length of life were dependent upon the survival of their loved ones or their ability to maintain or fulfill a particular life purpose. These findings corroborate apprehension expressed by Australians in a qualitative study investigating perceptions of life extension (Partridge et al., 2009) and young adults in Germany and the United States when considering more familiar forms of aging (Tahmaseb et al., 2003). Indeed, from a psychological perspective, the need to belong and to experience a sense of purpose and meaning in life would be critical concerns for the mental health and well-being of individuals with extremely extended life spans (Baumeister & Leary, 1995; Lansford, 2018; Maslow, 1968). There would, of course, be many benefits to an extended life span characterized by deep interpersonal connections without the decline of biological aging. If chronological aging, without nearing death, could be associated with meaningful work, as well as the established benefits of aging such as enhanced positive affect, emotional control, stability, and acceptance (Carstensen et al., 2011; Shallcross et al., 2013; Stone et al., 2010; Urry & Gross, 2010), then the appeal of living longer, from a psychological perspective, is readily apparent.

## Conclusion

The current work is the first systematic exploration of the mental characteristics associated with imagining life beyond the current life span. Three studies identified potential psychological hurdles of future life extension and charted the mental landscape of hypothetical aging scenarios. While future work may benefit from methodological approaches that extend beyond the single-item self-report measures utilized here, the current work provides a data-rich introduction to how different groups of individuals imagine life beyond the current life expectancy. Critically, this research also provides fodder for intriguing theoretical questions about the impact of age and hypotheticality on a sense of self-continuity in temporally distant simulations. Future research will be necessary to probe these factors and develop strategies to increase self-continuity in order to improve personal and environmental decision-making should the opportunity arise to extend life beyond the (current) life span.

## Funding

Funding for Mechanical Turk Participants was provided by Seattle Pacific University School of Psychology, Family and Community.
